# Treatment of digital ulcers in systemic sclerosis: recent developments and future perspectives

**DOI:** 10.1007/s10067-023-06511-0

**Published:** 2023-02-27

**Authors:** Ioannis Sagonas, Dimitrios Daoussis

**Affiliations:** 1 Rheumazentrum Ruhrgebiet, Ruhr-University Bochum, Herne, Germany; 2 Department of Rheumatology, Patras University Hospital, University of Patras Medical School, Patras, Greece

**Keywords:** Digital ulcers, Treatment, Scleroderma, Systemic sclerosis

## Abstract

Digital ulcers (DUs) comprise the main manifestation of vasculopathy and are a major cause of disability in patients with systemic sclerosis (SSc). A literature search in Web of Science, PubMed and Directory of Open Access Journals was performed in December 2022 to identify articles published in the last decade regarding the management of DUs. Prostacyclin analogues, endothelin antagonists and phosphodiesterase 5 inhibitors have shown promising results both as a stand-alone treatment and in combination for the treatment of existing and prevention of new DUs. Moreover, autologous fat grafting and botulinum toxin injections, although not readily available, can be of use in recalcitrant cases. Many investigational treatments with promising results could pave the way for a paradigm shift in the treatment of DUs in the future. Despite these recent advances, challenges remain. Better-designed trials are of paramount importance to optimise DU treatment in the years to come.

**Table:** 

**Key Points** • *DUs are a major cause of pain and reduced quality of life in patients with SSc.* • *Prostacyclin analogues and endothelin antagonists have shown promising results both as a stand-alone treatment and in combination for the treatment of existing and prevention of new DUs.* • *In the future, a combination of more powerful vasodilatory drugs, perhaps in conjunction with topical approaches, may improve outcomes.*

**Key Points**

• *DUs are a major cause of pain and reduced quality of life in patients with SSc.*

• *Prostacyclin analogues and endothelin antagonists have shown promising results both as a stand-alone treatment and in combination for the treatment of existing and prevention of new DUs.*

• *In the future, a combination of more powerful vasodilatory drugs, perhaps in conjunction with topical approaches, may improve outcomes.*

## Introduction

Systemic sclerosis (SSc), a prototype fibrotic disease, is characterised by an interplay between genetic predisposition and environmental triggers that leads to a dysregulated immune response, vasculopathy and eventually fibrosis. Among the various end-organ manifestations, digital ulcers (DUs) are one of the most common, affecting more than half of the patients at some stage during the course of disease, with the probability of developing digital ulcers reaching in some registries even 70% [1]. DUs are defined as well-demarcated areas of tissue loss of varying extent that can be denuded or covered by necrotic tissue [2]. From a pathophysiological perspective, fingertip DUs are thought to be a direct ischemic complication of the progressive vasculopathy, while those occurring over bony protuberances, mainly on the extensor aspect of the small joints of the hands, are most likely caused by recurrent microtrauma [3]. Albeit not the leading cause of morbidity and mortality in patients with SSc, DUs usually cause severe pain and disability due to functional impairment. DUs associate with a reduced quality of life and an increased economic burden, rendering their effective prevention and treatment a strategic target in SSc management [2].

The substantial progress of basic research in the last few years has led to a better understanding of the pathophysiology of SSc-related vasculopathy, identifying emerging therapeutic targets. In this narrative review, we highlight the most recent advances in the management of DUs in the past decade, including therapies currently in clinical trials or preclinical development. We also underline research priorities that could change the therapeutic landscape in the years to come.

## Methods

We performed an electronic search in Medline, Web of Science, Scopus and the Directory of Open Access Journals (DOAJ) from September 2012 to December 2022 using the following keywords: systemic sclerosis, digital ulcers and management in all combinations. We included cross-sectional studies, clinical trials, case series/reports and letters to the editor published in English language. Our search was focused only on articles related to the management of digital ulcers. A manual search was performed in the reference list of the included articles to extract relevant additional studies. Unpublished studies and gray literature were not considered.

## Results

### Pharmacological therapy

#### Prostacyclin analogues

Prostacyclin is a potent vasodilator with antithrombotic and antiproliferative properties that has been used in the treatment of refractory Raynaud’s phenomenon and SSc-associated DUs since the 1980s. The three prostacyclin analogues that are currently commercially available in Europe are iloprost, treprostinil and epoprostenol. Hypotension, headaches and flushing comprise some of their most encountered side effects and are mainly associated with the intravenous route of administration; however, a dose tapering to 0.5 ng/kg/min leads to a resolution of most adverse events in the majority of cases [4, 5]. BMI seems to be a major predictive factor of drug intolerance, as overweight patients tolerate lower iloprost infusion rates and have a 13-fold increased risk of developing adverse effects [6].

Iloprost is a synthetic analogue of prostacyclin that binds to prostacyclin and prostaglandin E2 receptors with equal affinity. Beside its antiplatelet, immunomodulating and cytoprotective properties, emerging data suggest a potential disease-modifying effect in patients with SSc [7]. Despite its short half-life, its clinical efficacy can extend for weeks after treatment cessation. This property may reflect its ability to inhibit CXCL10, a known, early contributor to SSc-associated vasculopathy, effectively preventing activation of endothelial cells and dermal fibroblasts in patients with SSc [8]. Moreover, iloprost stabilises endothelial adherence junctions preventing vascular dysfunction in SSc [9].

The PROSIT, an observational, multicentric study, retrospectively assessed an Italian cohort of 346 patients with SSc-related RP and/or DUs under long-term treatment with iloprost. For the management of active DUs, the authors employed and recommend a combination of iloprost plus calcium channel blockers (CCB) plus endothelin receptor antagonists. A reduction in the frequency and severity of DU lesions was reported by 74% of the participants using a validated Treatment Satisfaction Questionnaire for Medication. Most patients experienced relevant side effects such as hypotension, headache, flushing, vomiting and diarrhoea which were in only 14% of the cases prolonged. Some experts suggest the use of premedication like paracetamol or dopamine receptor antagonists to lower the rate of adverse events [10]. A similar retrospective real-world study found that 71% of patients with SSc receiving monthly iloprost infusions were free from DUs at the end of a decade-long follow-up period [11]. On the other hand, iloprost withdrawal was linked to worsening of RP and DUs recurrence [12]. Most patients on monthly iloprost infusions experience complete clinical resolution in the first year of treatment with the 10-year survival rate reaching in one cohort 55.6% [13]. In the absence of tools such as capillaroscopy, which require a certain level of experience and are operator dependent, simple clinical tests such as Allen’s test that can be performed easily at bedside can be employed as predictors of patient outcome; DUs in patients with a negative Allen’s test show expedited healing times [14]. Clinical improvement and a switch to easier, more cost-efficient ways of administration such as an elastomeric pump seem to be the main reasons of drug discontinuation [13]. Elastomeric pumps have been shown to be equally effective to the intravenous route, provide greater patient autonomy and are associated with fewer adverse events because of their continuous slow-release rate [15].

Despite the central role of iloprost in the management of DUs, its optimal infusion schedule has yet to be established. High-quality data from randomised trials are lacking, as all studies published to date assessed only short treatment regimens, no more than 5 days of daily treatment. In a recently published French observational retrospective study, a longer duration of iloprost treatment (more than 5 days, median 7.3 days) shortened DU healing times by half in comparison to shorter 3–5-day treatment courses (48 [7–392] vs. 91 [9–365] median days, range, respectively). At 3 months, more than half of the patients treated with the prolonged duration regimen experienced a complete resolution of the DUs in contrast to a mere 37% in the other cohort. The number of active DUs before treatment, concurrent treatment with endothelin antagonists, calcium channel blockers or other immunosuppressive agents, such as cyclophosphamide and methotrexate, did not affect healing times. Moreover, a statistical significance could not be observed in the number of DU-related complications between the two groups. The observed side effects were more common in patients receiving concomitantly calcium channel blockers [16]. Collectively, longer treatment courses could represent an efficacious, albeit costly alternative, especially in patients with more severe disease.

Treprostinil, a newer oral prostacyclin analogue, did not reach the prespecified primary endpoint of overall DU burden reduction after 20 weeks of treatment in a large, double-blind placebo-controlled trial (DISTOL-1). Nevertheless, a retrospective analysis of the medical charts of the patients after the termination of the trial as well as unpublished data from the extension study that succeeded it found evidence of a significantly increased total DU number following discontinuation of treprostinil and after adjusting for potential seasonal effects. Most of the observed side effects are dose-dependent, of mild or moderate intensity and include headache, diarrhoea and nausea. The failure to meet the primary endpoint in the randomised trial could be attributed to a heterogeneity of the initial patient cohort or inherent difficulties in performing high-quality DU studies such as the variable, experience-dependent definition of DU activity [17, 18]. Further study is warranted, when feasible taking into account specific biomarkers such as the subtype of SSc and the antibody status of the patients; patients with diffuse cutaneous SSc and no evidence of anticentromere antibodies may profit from treatment with treprostinil. Table 1 summarises all key data regarding prostacyclin analogues.

**Table 1:** Studies assessing the efficacy of prostacyclin analogues

Author/year (ref.)	Study type	SSc type	No. of participants	DU definition	No. of patients with DUs at baseline	Intervention	Outcome
Negrini et al./2019 [10]	Observational, retrospective study	dSSc: 80, lSSc: 238, SSc sine scleroderma: 15, early scleroderma: 13	346	D1	159	Iloprost: 0.5 to max. tolerated doses not exceeding 2.0 ng/kg/min over 4 to 8 h, iloprost induction: 1 to 5 consecutive days Maintenance: 1 day/4 weeks	74% of the patients reported a reduction in the frequency of DUs.
Colaci et al./2017 [11]	Observational, retrospective study	dSSc:13, lSSc:37	50	D1	31	Monthly infusion at 0.8–1 ng/kg/min (average cumulative dose 25 μg)	71% of the patients were free from DU during the 10-year follow-up. No severe side effects reported.
Martins et al./2022 [13]	Observational retrospective study	dSSc:9, lSSc: 37	60	N/A	44	Monthly infusion at 0.5–1.5 ng/kg /min, infusion rate as tolerated starting at 4 mL/h and increased by increments of 4 up to 16 mL/h	86.4% of patients: DUs healing in the first 6 months Safety: generalised erythroderma in one patient, others with minor AEs, 5-year survival rate: 68.2%
Casigliani et al./2012 [14]	Observational retrospective study	dSSc: 31, lSSc: 42	73	N/A	28	Monthly infusion at 0.5–1.5 ng/kg /min, infusion rate as tolerated	Complete healing in 89.4% of the patients after the first treatment
Jamart/2022 [16]	Observational longitudinal retrospective study	dSSc:10, lSSc: 31	41	D2	13	Iloprost Infusion > 5 days vs. < 5 days Median infusion rate of 1.7 ng/kg/min.	Number of patients with complete DU healing at day 90 was significantly higher among patients who received > 5 days of iloprost: 51 vs. 37% (*p* < 0.05).
Shah et al./2016 [17]	Retrospective study based on data from DESTOL-1 and DESTOL-EXT	dSSc: 8, lSSc:43	51	D3	N/A	Oral treprostinil twice daily	Increase in DU burden after discontinuation of oral treprostinil

*dSSc* diffuse SSc, *lSSc* limited SSc

D1: loss of epidermal covering with a break in the basement membrane

D2: a lesion with visually discernible depth and a loss of continuity of epithelial coverage, which could be denuded or covered by a scab or necrotic tissue, localised at distal to the proximal interphalangeal joints and without bone infection or calcinosis

D3: area with clearly discernible depth and a loss of continuity of epithelial coverage, which could be denuded or covered by a scab or necrotic issue

In addition to prostacyclin analogues, selexipag, an oral selective IP-prostacyclin receptor agonist, which is currently approved for the treatment of pulmonary hypertension, led to complete healing of the DUs in 6 patients with SSc, after first- and second-line agents failed to show efficacy. The observed effect was achieved after 3–6 months of treatment with 2.400–3.000 mg selexipag daily [19]. Despite these promising results, clinical trial data to support the routine use of selexipag in hard-to-treat DUs are lacking.

#### Endothelin antagonists

Endothelin-1 (ET-1), a potent vasoconstrictive and potentially inflammatory and fibrotic mediator, is considered a key orchestrator of the vascular changes and tissue remodelling in SSc. Although bosentan, ambrisentan and macitentan are currently used for the treatment of pulmonary arterial hypertension, only bosentan, a competitive antagonist targeting both ET_A_ and ET_B_ endothelin receptors, is currently licenced for the prevention of DU development.

Two randomised double-blind placebo-controlled trials provided rationale for its use in DU treatment. Most recently, in the RAPIDS-2 trial, 188 patients with SSc and at least one active DU were enrolled in a 20-week comparison of 125 mg bosentan twice daily versus placebo, after the patients were treated with half the dose of bosentan for 4 weeks at the initial phase of the study. A substantial reduction of 30% in the development of new DUs in patients with both diffuse and limited SSc was observed [20], although, in a subsequent study, no relationship could be established between this favourable outcome and the bosentan-induced increased digital blood flow [21]. A wide individual variability in the hand blood flow of patients with SSc could account for this lack of association. This beneficial effect was not extrapolated in the times to healing of the active DU, patient-reported overall hand pain scores or ulcer burden, as no differences were evident between the treatment and placebo group [20]. Even though the overall number of side effects did not differ significantly between the two groups, the increased incidence of elevated liver enzymes in subsects receiving treatment highlights the need for regular blood monitoring in patients on bosentan.

An Italian retrospective case-control study further corroborated a statistically significant lower occurrence of DUs in bosentan-treated patients with SSc [22], while the beneficial effects of bosentan were further reflected by an improvement of the self-reported visual analogue scale-digital ulcers score at a 12th-month follow-up visit, in another study [23]. In a subsequent prospective observational study in a Turkish cohort with SSc, 26.7% of the patients with diffuse cutaneous SSc developed new DUs under bosentan, while four patients suffered from critical digital ischemia requiring additional treatment with iloprost [24].

In other reports, bosentan was shown to be effective in the management of nondigital ulcers too, specifically those occurring on the basis of an impaired peripheral circulation [25, 26].

Moreover, mounting data provide evidence for the use of bosentan as an add-on treatment in patients with SSc already on iloprost. In a retrospective study of 34 patients with SSc and refractory digital ulcers despite 6 months of iloprost treatment, the addition of bosentan in the therapeutic regimen led to a significant decrease in the mean number of digital ulcers on the hands from 1.7 to 0.7 (*p* = 0.00003). A similar effect could not be observed in regard to the lower limbs. The degree of digital skin fibrosis seems to play a pivotal role in DU healing since only 18% of ulcers in patients with severe digital fibrosis healed in comparison with 80% in patients with mild disease [27]. Furthermore, in an Italian study of 30 patients with SSc, this synergistic effect was reflected by a statistically significant reduction of 80% in the incidence of new DUs [28], while a more recent retrospective study showed a 37.84% decrease in the prevalence of DUs with concurrent treatment [29]. An accompanying increase of capillaries acting as a surrogate marker for an improvement of the microvascular damage has also been reported under ET-1 antagonism and iloprost combination [28, 30]. Finally, bosentan seems to be a promising treatment for DUs presenting in the context of a paraneoplastic syndrome [31].

Macitentan is, like bosentan, a dual endothelin ET_A_/ET_B_ receptor antagonist. Due to its slower receptor dissociation rate, macitentan possesses a theoretical potential to block ET-1 signalling more effectively than other ET-1 inhibitors [32]. In the phase III randomised, double-blind, placebo-controlled DUAL-1 and DUAL-2 trials, which involved approximately 70 centres worldwide each, the effectiveness of macitentan was evaluated in 226 and 216 patients, respectively, with SSc and active DUs. In each study, patients were either treated with 3 mg or 10 mg of macitentan or placebo once daily. Both trials could not achieve the primary endpoint of a reduction in the cumulative number of new DUs at week 16 of follow-up. Moreover, macitentan failed to improve hand function or reduce the overall hand pain related to DUs. These disappointing results led to a premature termination of DUAL-2, a decision based on patient safety concerns [33]. However, the findings of the DUAL trials should be interpreted critically. Better patient and clinician’s education as well as easier access to online information material on the prevention and management of SSc-associated DUs may be held accountable for the observed decrease in the number of new DUs in the control groups of both studies in comparison with the RAPID-2, thus rendering a possible beneficial effect of macitentan undetectable. Furthermore, the prior use of prostanoids and bosentan for the treatment of DUs might have posed a barrier regarding recruitment in the DUAL studies, excluding patients with severe DUs, who at least theoretically would be more likely to benefit from treatment. Despite some recent positive case reports [34, 35], macitentan is not yet a recommended treatment for DUs in the updated EULAR guidelines for the treatment of SSc.

Ambrisentan, a highly selective ET_A_ inhibitor, has been shown to abate cellular proliferation and vasoconstriction while maintaining the vasodilatory effects of ET_B-_mediated signalling [32]. In a case series of six patients with SSc-related DUs receiving intravenous prostanoids, who were previously unsuccessfully treated with bosentan as an add-on therapy, ambrisentan administered at a dose of 5 mg/day resulted in the complete healing of all DUs in four patients at the end of the 24-week observation period. No new ulcers were detected while the number of RP attacks decreased significantly in all participants (Δ −3.10 *p* = 0.01) [36]. A larger, prospective open-label study found a similar reduction in the total number of DUs per patient (from 3.1 ± 2.1 to 1.3 ± 1.6, *p* = 0.004, weeks 0 and 24, respectively). However, ambrisentan did not prevent the development of new DUs over the study course [37]. A subsequent 12-week RCT found no improvement in the digital microvascular blood flow of patients treated with ambrisentan, indicating no measurable vasodilatory effect [38].

#### JAK inhibitors

Small molecules that inhibit the JAK signalling proteins are gaining traction in the past 5 years as treatment options in a wide range of rheumatic diseases. In SSc, Janus kinases are important transducers of pro-inflammatory and pro-fibrotic signals to key players of SSc pathogenesis, including fibroblasts and endothelial cells. In a case series, 3 out of 4 female patients with diffuse SSc and active DUs at baseline experienced a complete ulcer resolution at week 24 of treatment with baricitinib, while no new ulcers developed in any of the 10 patients treated. Furthermore, baricitinib led to a significant mean improvement of 49.23% in the Rodman skin score of the patients. The underlying pathophysiologic mechanisms that mediate this effect remain to be elucidated [39]. Tofacitinib was also used successfully in the treatment of DUs in an African American male patient with diffuse SSc [40]. Taken together, these data may pave a promising future for JAK inhibitors in SSc-associated DUs.

### Topical treatment

#### Autologous fat grafting and mesenchymal cells transplantation

Accumulating evidence suggests that regional implantation of autologous adipose tissue-derived cell fractions could be a viable option for recalcitrant DUs. Adipose-derived stromal/stem cells (ASCs) exhibit an immunosuppressive capacity and angiogenic properties similar to those of mesenchymal stromal cells (MSCs) derived from bone marrow while they are easier to isolate and linked to reduced donor morbidity [41]. Based on the encouraging results of previous pilot studies [42, 43], an Italian group performed a monocentric RCT where autologous adipose tissue and placebo were injected at the base of the affected finger in 25 and 13 patients with active SSc-related DUs, respectively. Treatment with intravenous prostanoids and calcium-channel inhibitors, initiated before inclusion in the study, was continued in all patients enrolled. Complete DU healing was achieved in 92% of the patients treated with adipose tissue grafting after 8 weeks, while only one patient in the placebo arm experienced improvement of the DU (*p* < 0.0001). Moreover, no new ulcers had appeared in any of the patients treated with adipose tissue at a 3-month follow-up visit [44].

In a single-centre, open-label pilot study, autologous MSCs were injected in 40 patients with ischemic DUs due to either SSc or arteriosclerosis-associated peripheral artery disease (11 and 29 patients, respectively). Even though the visual analogue pain scores decreased significantly after treatment, 18.2% of the patients with SSc suffered from a recurrence of limb ischemia at the 2-year follow-up, while one patient required digital amputation [45].

#### Botulinum toxin injection

Several case series provide evidence for the use of botulinum toxin A (BTX-A) in the treatment of intractable DUs [46–49]. BTX-A, a selective acetylcholine release inhibitor, is thought to improve digital blood flow and inhibit vasoconstriction via vascular smooth muscle paralysis and blocking of noradrenaline release [47]. A recent study showed that a higher concentration and total dose of BTX-A when injected at the digital neurovascular bundle may lead to improved outcomes, such as vasospastic symptom control and ulcer healing with a favourable adverse effect profile [49]. In a subsequent single-blind RCT from the same group, BTX-B was shown to be effective in accelerating the healing of refractory to standard treatment DUs and preventing the development of new ones at 16 weeks of follow-up in patients with SSc [50]. Moreover, topical BTX treatment seems to be a cost-efficient, equally effective alternative to intravenous prostanoids that can be administered in the outpatient setting [51]. Collectively, more clinical data are needed to establish botulinum toxin injections as a standard treatment for DUs.

#### Sympathectomy

Although thoracic sympathectomy is nowadays an obsolete method owing to a high rate of adverse events, digital sympathectomy may represent a viable option for DUs refractory to standard care. A retrospective study of SSc-related DUs found a 92.3% post-sympathectomy improvement in pain, while all but two patients experienced DU healing [52].

### Investigational treatments options

#### Riociguat

Riociguat, a first-in-class guanylate cyclase stimulator, is currently approved for the treatment of pulmonary arterial hypertension and chronic thromboembolic arterial hypertension. A randomised, double-blind, placebo-controlled trial did not find any difference of statistical significance in the net ulcer burden (primary endpoint) and other secondary outcome measures such as healing of all baseline DUs and healing of the cardinal DU between the 2 groups at week 16 of treatment [53]. However, a longer treatment duration might have been necessary for the treatment effect to be notable, as suggested by the positive trends in DU healing, documented in the open-label phase of the study.

#### Ozone

Ozone, a bactericidal gas with antioxidant properties and proven efficacy in the management of chronic wounds, was found in a randomised, blinded, controlled trial of 50 female patients with SSc-associated DUs to induce a significantly greater ulcer size reduction in comparison to calcium channel blockers (control group) at day 20 of treatment. The ulcer pain score and the number/duration of Raynaud’s phenomenon attacks were also decreased in the ozone treatment arm. The reported upregulation of growth factors such as VEGF and TGFβ in local ulcer tissue after ozone treatment may promote epithelialization and at least partially account for the observed effect [54]. A subsequent smaller study yielded similar results [55].

#### Hyperbaric oxygen therapy

Hyperbaric oxygen therapy (HBOT) utilises pure oxygen applied in a closed chamber at increased pressure, generally 2–3 atmospheres to induce hyperoxia and hyperoxemia. Its proven antimicrobial, angiogenic and immunomodulatory effects are used therapeutically in a wide array of diseases, including chronic ulcers [56]. In a recent report, the DUs in 2 out of 3 female patients with SSc healed completely, after 60 and 40 HBOT therapy sessions, respectively. No adverse events were documented during the treatment [57]. The therapeutic effects are thought to be mediated via an enhanced oxygen delivery in the DUs’ hypoxic environment and an upregulation of NO production, a potent vasodilator. Another case study provided similar results [58].

#### Extracorporeal shock wave therapy

Extracorporeal shock wave therapy (ESWT), a technique primarily used in lithotripsy, is recently shown to accelerate tissue regeneration and promote angiogenesis. In a phase 2 pilot study of 9 patients with SSc-related DUs, ESWT was effective in reducing DU size and number, although it did not prevent the development of new ones. Treatment was not associated with any side effects [59].

#### Low-level light therapy

Light treatment has been lately successfully employed in the treatment of recalcitrant skin ulcers. The therapeutic effect is thought to be achieved through a plethora of mechanisms, including stimulation of collagen deposition, an antibacterial effect and a local NO-dependent increase in tissue perfusion. In a feasibility study, a novel combination of infrared, red and violet light showed promising results as a safe, minimally invasive treatment in the management of DUs [60]. Currently, an ongoing, open-label, prospective randomised controlled trial (S.U.I.T.A.B.L.E) will assess the clinical effectiveness of a portable blue light device on DU healing after 16 consecutive weeks compared to standard treatment in patients suffering from SSc.

#### Rheopheresis (RheoP)

RheoP is a safe and effective therapeutic modality to treat microcirculatory disorders. It comprises a double filtration plasmapheresis system that selectively removes large plasma proteins, thus reducing the whole blood viscosity. RheoP was successful in treating recalcitrant DUs in a female patient with SSc. No new ulcers were reported during treatment and at a 3-month follow-up visit. Moreover, no major side effects were seen. To further investigate the potential benefit of Rheopheresis in Raynaud’s phenomenon and DUs, the Rheact, a randomised controlled feasibility study, is currently ongoing [61, 62] (Trial Identifier: NCT05204784).

#### G-CSF

Granulocyte colony-stimulating factor (G-CSF) is an immunoregulatory cytokine that exerts antimicrobial, angiogenetic and tissue regenerative effects. Daily treatment with filgrastim 300 μg, a G-CSF analogue, over three consecutive days resulted in a complete resolution of DUs in eight out of ten patients with SSc, while the mean time to DU healing was 1 and a half months [63].

#### Phosphodiesterase 5 inhibitors (PDEi5)

PDEi5 inhibit phosphodiesterase, an enzyme that catalyses the hydrolysis of cGMP in endothelial cells. The resulting increase in cGMP levels induces muscular smooth muscle relaxation and vasodilatation [64]. Because of these properties, they are recommended as a treatment for DUs in the updated EULAR guidelines for the management of SSc. Adverse events are not uncommon and include vasomotor reactions, myalgias, nasal stuffiness, visual abnormalities and allergic reactions [65].

Sildenafil, used primarily for the treatment of pulmonary hypertension in SSc at 20 mg three times daily orally, decreases the severity, frequency and duration of RP attacks. In a randomised, double-blind, placebo-controlled trial (SEDUCE trial), 83 patients with SSc were randomly assigned to either sildenafil 20 mg thrice daily or placebo. Sildenafil reduced the number of DUs per patient at weeks 8 and 12 (1.23 ± 1.61 vs. 1.79 ± 2.40, *p* = 0.04; 0.86 ± 1.62 vs 1.51 ± 2.68, *p* = 0.01, in the sildenafil and placebo group, respectively). Even though the primary endpoint evaluating the time to DU healing was not achieved because of a higher-than-expected healing ratio in the placebo group, sildenafil remains a useful treatment option for DUs in SSc [66].

Tadalafil, a phosphodiesterase 5 inhibitor, was recently found to be effective in reducing the RP-associated pain when used as an add-on topical therapy to standard care. Although a significant decrease in the number of DUs was not achieved, larger, randomised trials are needed to draw definitive conclusions [67].

Other treatments that were found to be effective in DU management include low-dose immune globulin and oral psoralen plus ultraviolet A therapy [68, 69]. The above investigational agents are summarised in Table 2.

**Table 2:** Studies assessing the efficacy of investigational agents in the treatment of DUs

Author/year (ref.)	Study type	SSc type	No. of participants	DU definition	No. of patients with DUs at baseline	Intervention	Outcome
Nacaraya et al./2019 [53]	RCT	dSSc: 8, lSSc: 9	17	D4	17	Riociguat (9 patients) vs. placebo (8 patients)	No significant difference in the total DU burden in the two groups
Hassanien et al./ 2018 [54]	RCT	dSSc: 19, lSSc: 31	50	D5	50	Ozon therapy (plus CCB vs. CCB alone)	Healing rate higher in ozone group than in placebo group [96% vs. 44% (*p* < 0.007)]
Mirasoglu et al./2017 [57]	Case series	dSSc: 3, lSSc: 2	6	N/A	3	53 mean HBOT sessions; one session per day, 5 days a week, each session of HBOT lasted about 120 min	Complete healing of DUs in 2/3 patients.
Gerodimos et al./2013 [58]	Case report	lSSc: 1	1	N/A	1	34 HBOT sessions at 244 kPa (2.4 ATA) for 90 min plus daily ulcers debridement	Improvement of the DU
Shaito et al./2015 [59]	Case series	dSSc: 5, lSSc: 4	9	N/A	9	ESWT: 100 pulses at 0.08–0.25 mJ/mm^2^ in 20 areas on both hands and 15 areas on both feet, totaling 7000 pulses once weekly for 9 weeks	Decrease in the mean number of DUs per patient
Hughes et al./2019 [60]	Case series	dSSc: 3, lSSc: 4, SSc-spectrum disorder: 1	8	N/A	7	Irradiation with light therapy 10 J/cm^2^ twice weekly for 3 weeks	Improvement in DU status during treatment assessed by clinician and patients
	Case report	dSSc	1	Current definition of DU proposed by World Scleroderma Foundation	1	Rheopheresis 2×/weekly every 4 weeks for 3 months.	Clinical improvement of current DUs, no new DUs while on treatment
Keret et al./2022 [63]	Case series	dSSc: 5, lSSc: 5	10	N/A	10	Filgrastim 300 mcg over 3 days	Complete resolution of DUs in 80%, reduction in DU number from 2.23 ± 2.20 to 0.50 ± 0.67at 3 months (*p* < 0.05)
Hachulla et al./2016 [66]	RCT	dcSSc: 39, lSSc: 7, lcSSc: 37	83	D6	83	Sildenafil 20 mg or placebo 3×d. for 12 weeks	Significant decrease in DU number at weeks 8 and 12.
Fernández-Codina et al./2020 [67]	Retrospective observational study	dSSc: 5, lSSc: 7, sine: 1	135	N/A	13	10 mg tadalafil cream 2×d. as add-on to standard treatment for 4 weeks	Reduction of DU number close to statistical significance
Perkovic et al./2020 [68]	Retrospective observational study	Diffuse: 8, limited: 1	9	N/A	6	IVIG	Complete DU healing
Inoue T et al./2014 [69]	Case report	dSSc	1	N/A	1	PUVA 3 times/week for 4 weeks, cumulative dose 23 J/cm^2^	Ulcer healing and MRSS improvement from 32 to 15.

*ESWT* extracorporeal shock wave therapy, *HBOT* hyperbaric oxygen therapy, *dSSc* diffuse SSc, *lSSc* limited SSc

D4: a full-thickness skin lesion, > 3 mm in maximal diameter, with loss of epithelisation, epidermis and dermis

D5: a loss of epithelisation and tissue, involving in different degrees the epidermis, the dermis, the subcutaneous tissue and sometimes also involving the bone

D6: a break in the skin with a loss of epithelisation on the distal finger surface of ischaemic origin according to the physician and not located over subcutaneous calcifications or over extensor surfaces of joints.

## Discussion

Even though DUs are not directly linked to the increased mortality seen in patients with SSc, they are very important from a clinical point of view since they associate with excruciating pain and significant functional impairment. The treatment of DUs remains challenging and suboptimal despite recent advances. In everyday clinical practice, the endothelin antagonist bosentan is commonly used for the prevention of new DUs. However, its high cost is a significant burden especially in countries with strict health care budget, but this issue may improve in the near future with the wider use of generics. There are only very limited data regarding the efficacy of other endothelin antagonists, and therefore, their use should be restricted to selected cases. On the other hand, prostacyclin analogues are certainly one of the most effective agents for the prevention and treatment of DUs, but again, their use has limitations such as very high cost, low availability and problematic route of administration. A combination of bosentan and iloprost appears promising for difficult-to-treat cases. Apart from these drugs, one should keep in mind that simple nonpharmacologic measures such as smoking cessation and avoiding exposure to cold are of outmost importance. All physicians should strongly encourage patients with SSc to apply these simple but critical lifestyle changes. Topical treatment in the form of autologous adipose grafting and botulinum toxin have shown promising yet preliminary results, and for the time being, they should be used with caution in selected cases in highly specialised centres.

In conclusion, during the last decade, we had some significant advances such as the approval of bosentan for the prevention of DUs, but still, we have a long way to go. There are several treatment options, but so far, efficacy has been modest. We definitely need more large-scale studies assessing combination treatments such as iloprost plus endothelin antagonists or endothelin antagonists plus PDE5i. In the future, a combination of more powerful vasodilatory drugs, perhaps in conjunction with topical approaches, may improve outcomes.
